# Seeing Through Packaging: Eye-Tracking Evidence on How Product Visual Strategy and Unit Size Shape Visual Attention and Consumer Evaluation

**DOI:** 10.3390/jemr19020030

**Published:** 2026-03-10

**Authors:** Zhiyi Guo, Zihao Cao, Yongchun Mao, Muhizam Mustafa, Yuqi Luo, Yueyue Ning

**Affiliations:** 1 School of the Arts, University Sains Malaysia, Gelugor 11800, Penang, Malaysia; zhiyi@student.usm.my (Z.G.); mmuhizam@usm.my (M.M.); luoyuqiyuki@student.usm.my (Y.L.); 2 School of Design and Architecture, Zhejiang University of Technology, Hangzhou 310014, China; zeno@zjut.edu.cn; 3 School of Arts and Design, Qilu University of Technology, Jinan 250353, China; ycmao@qlu.edu.cn

**Keywords:** eye tracking, visual attention, product visual strategies, transparent packaging, food unit size, purchase intention

## Abstract

Product visual strategies (PVS) on food packaging influence how consumers visually inspect products at the point of purchase. However, evidence comparing transparent windows and product images remains mixed, particularly regarding how these strategies interact with food unit size (FUS) and shape visual attention patterns. Moreover, few studies have examined these effects using objective eye-tracking measures within controlled experimental designs. This study employed a 2 × 2 between-subjects quasi-experiment to investigate the effects of PVS (transparent window and product image) and FUS (large unit and small unit) on visual attention and subsequent product-related evaluations. A total of 160 participants viewed realistic chocolate package stimuli that varied only in visual strategy and unit size. Eye movements were recorded using Tobii Pro Glasses 2. Visual attention was assessed through Time to First Fixation (TFF) and Fixation Duration (FD), while expected tastiness, expected quality, and purchase intention were measured using standardized self-report scales. The results showed that transparent-window packaging attracted visual attention more rapidly and sustained longer fixations than product-image packaging. These attention differences were accompanied by higher expected tastiness, expected quality, and purchase intention. While food unit size alone showed limited effects on eye-movement measures, a significant interaction was observed: small-unit designs elicited greater visual attention and more favorable evaluations only when the product was directly visible through a transparent window. Overall, the findings demonstrate how product visual strategies and food unit size jointly shape visual attention allocation during packaging inspection. By integrating eye-tracking measures with evaluation and behavioral intention outcomes, this study contributes to applied eye-movement research in food packaging contexts.

## 1. Introduction

Food packaging has become an indispensable part of daily life, serving as a container for storing products and fulfilling functions of protection, transportation, convenience, and communication [1]. However, beyond its basic functions, packaging now increasingly showcases product features to attract consumers’ attention and perception, encouraging purchases [2]. Because packaging plays a crucial role in product marketing, it can influence consumers’ perceptions of packaged products and, consequently, their purchasing behavior [3]. The visual stimuli, informational elements, and functional attributes of food packaging can help consumers judge the expected tastiness and quality of the food, thus influencing their decision-making [4,5]. Therefore, food packaging design typically integrates material and visual aspects. The material dimension involves the selection of packaging materials (such as paper and paper-based materials, plastic films, glass, or multilayer barrier structures) as well as production techniques such as printing, lamination, and surface finishing processes. These material and processing choices affect not only the functional performance of packaging, such as product protection, barrier properties, hygiene, and shelf-life stability, but also influence consumer perceptions of quality, safety, and environmental sustainability, thereby shaping purchase decisions [6,7,8,9]. In contrast, the visual dimension includes key elements such as graphics, color, shape, and labeling, which function as extrinsic cues that attract attention and influence consumer perception and evaluation at the point of purchase [10,11,12,13].

In recent years, transparent window design has emerged as a prominent trend in food packaging, representing a crucial element of product visual strategy (PVS) [14]. By allowing consumers to see the actual product, transparent packaging strengthens perceptions of authenticity, which encompass key attributes such as product quality, healthiness, and freshness, ultimately shaping purchasing behavior [15,16,17]. However, traditional non-transparent packaging designs remain widely used in the food industry, particularly those featuring product images [18]. This may be because such images effectively attract consumers’ visual attention and shape their expectations of the product’s sensory attributes, thereby enhancing their overall product evaluation and influencing their purchasing decisions [19]. Nevertheless, compared with traditional non-transparent packaging, the effects of transparent window designs on consumers’ perception and purchase intention are still not well understood, highlighting the need for further research on this topic [18,19]. However, transparent packaging may not be suitable for all product categories. For light-sensitive or high-fat products (such as fried snacks and dairy products), exposure to light may accelerate photo-oxidation, leading to quality deterioration and reduced shelf life [20]. In such cases, opaque or light-barrier packaging is often preferred to ensure product stability.

Several studies in the literature have examined the effects of transparent packaging and product imagery on consumer behavior, including product evaluation and purchase intention. For example, Deng and Srinivasan [21] conducted five experimental studies to investigate how transparent packaging influences food consumption and proposed two opposing mechanisms: the salience effect (which increases consumption) and the monitoring effect (which decreases consumption). Their findings suggest that transparent packaging does not always promote consumer purchase or consumption. Specifically, transparency tends to increase consumption for small, visually appealing foods such as M&M’s, but it reduces consumption for larger foods such as cookies and for healthy foods such as vegetables. It is important to note that in this study, the definition of food unit size was not based on portion size, but rather on the “unit of consumption” derived from actual eating behavior. For instance, participants typically consumed small-unit foods piece by piece (one candy at a time), whereas large-unit foods were eaten in larger chunks, with each piece counted as one unit of consumption [21].

In addition, Simmonds et al. [14] investigated the effects of transparent and traditional non-transparent packaging on consumers’ product perception and purchase intention. Their experiments demonstrated that transparent window designs generally enhanced consumers’ expectations of tastiness, freshness, quality, and purchase intention across several product categories, including cereals, chocolates, dry pasta, and salmon. Although the study found that transparent window designs were more effective than product images in increasing purchase intention, it did not examine whether the direction of this effect varied according to food unit size. However, an eye-tracking study later challenged this assumption by showing that transparent packaging does not necessarily attract more attention or induce higher purchase intention than product image packaging. The study found that food type and the aesthetic quality of the packaging could both influence product attention and purchase intention [18]. Its experimental data indicated that, in most cases, regardless of food unit size, transparent window designs and product images were similarly attractive to consumers.

Although prior research has examined how transparent windows and product images influence consumer perceptions and purchase intentions [5,14,18,19,21], most existing studies rely primarily on self-reported evaluations and behavioral outcomes. The underlying visual attention mechanisms through which these packaging strategies operate remain insufficiently understood. In particular, there is limited eye-tracking evidence explaining how product visual strategy (PVS) shapes attentional allocation, and whether intrinsic product characteristics such as food unit size (FUS) moderate these effects under controlled experimental conditions. Furthermore, the linkage between objective attention measures and subsequent product evaluation has not been systematically examined. To address these gaps, the present study investigates how PVS (transparent window vs. product image) and FUS (large vs. small unit) jointly influence visual attention, product evaluation, and purchase intention using a controlled eye-tracking design. Drawing on Cue Utilization Theory and visual attention research, the study examines how intrinsic and extrinsic cues interact to shape attentional orientation and processing depth. Accordingly, this study is guided by the following research questions:

RQ1: How does product visual strategy (transparent window vs. product image) influence visual attention allocation, as measured by TFF and FD?
RQ2: Does food unit size moderate the effect of product visual strategy on visual attention?
RQ3: How do visual attention patterns relate to expected tastiness, expected quality, and purchase intention?

Cue Utilization Theory states that consumers use both intrinsic and extrinsic cues to evaluate product quality during decision-making [22]. Ingredients, size, and taste are usually considered intrinsic cues (physical attributes that are not easily changed), while brand, price, country of origin, and packaging are usually considered extrinsic cues (not part of the product itself but still important for consumer perception) [23,24]. Existing studies show that when product quality is uncertain, consumers often rely on extrinsic visual cues from packaging to form initial judgments, which then shape their product perceptions [7,25,26]. Expected tastiness and expected quality are important motivations for food choice, and previous empirical work has shown that both are related to consumer attitudes and purchase intentions [19,24,27,28]. At the same time, visual attention is a necessary condition for consumers to receive these cues and plays an important role in how consumers process information [29,30]. In this study, FUS is an intrinsic cue because it is a feature of the product itself. Product images are extrinsic cues because they do not directly show the actual product. Interestingly, transparent packaging has a dual function. On one hand, it shows the actual product, allowing consumers to perceive intrinsic attributes such as size. On the other hand, as part of packaging design, it also attracts attention from the outside [19]. Thus, in real applications, transparent packaging can serve as both an intrinsic cue and an extrinsic cue, carrying information while attracting visual attention. Based on Cue Utilization Theory, these cues may influence consumers’ visual attention and their perceptions, such as expected tastiness and expected quality, which are related to their purchase intentions.

In this study, food unit size is regarded as an intrinsic cue because it represents a physical characteristic of the product itself, whereas product images are considered extrinsic cues, as they are not directly related to the product. Interestingly, transparent packaging plays a dual role in consumer perception. On the one hand, it enables the visibility of the product’s physical attributes, such as size, allowing consumers to directly perceive intrinsic qualities. On the other hand, as part of the packaging design, it attracts consumers’ attention through its appearance [19]. Therefore, transparent packaging can be regarded as both an internal cue and an external cue, playing the dual role of conveying product information and being visually appealing. Based on the cue utilization theory, these different types of cues can influence consumers’ visual attention and their perceptions of taste and quality, which ultimately shape their purchase intentions.

## 2. Materials and Methods

### 2.1. Research Design

To address the research questions, this study adopted a quasi-experimental design. An eye-tracking device was used to capture and record participants’ physiological responses during their visual interactions with chocolate packaging. Moreover, a questionnaire was used to assist in collecting quantitative data on the participants’ subjective evaluations.

The experiment employed a 2 (PVS: transparent window and product image) × 2 (FUS: large unit and small unit) design. The dependent variables included participants’ subjective evaluations of Expected Tastiness, Expected Quality, and Purchase Intention, as well as eye-tracking–based measures of visual behavior, namely Fixation Duration (FD) and Time to First Fixation (TFF). FD refers to the total time (in milliseconds) that a participant’s gaze remains fixed on a defined area of interest (AOI), reflecting the depth of visual processing. TFF refers to the time elapsed from stimulus onset until the participant first fixates on a predefined AOI, indicating the speed of attentional orientation [31,32].

FD and TFF were selected because they capture complementary components of visual attention that are theoretically relevant to packaging cue processing. According to the Eye–Mind Hypothesis [33], eye movements are closely linked to underlying cognitive processing, such that fixation patterns reflect moment-to-moment information processing. In this context, TFF indexes early attentional capture and orienting toward a visual cue, whereas FD reflects sustained attention and depth of processing after attention has been allocated [31,32]. Together, these metrics provide an objective basis for examining how packaging cues influence cognitive evaluation and purchase-related judgments.

The entire implementation process was conducted from 15 May 2025 to 10 September 2025, covering key phases such as stimulus development, participant recruitment, data collection, and data analysis. Throughout this period, all activities were carried out systematically and in accordance with ethical standards.

### 2.2. Stimuli

**Figure 1:**
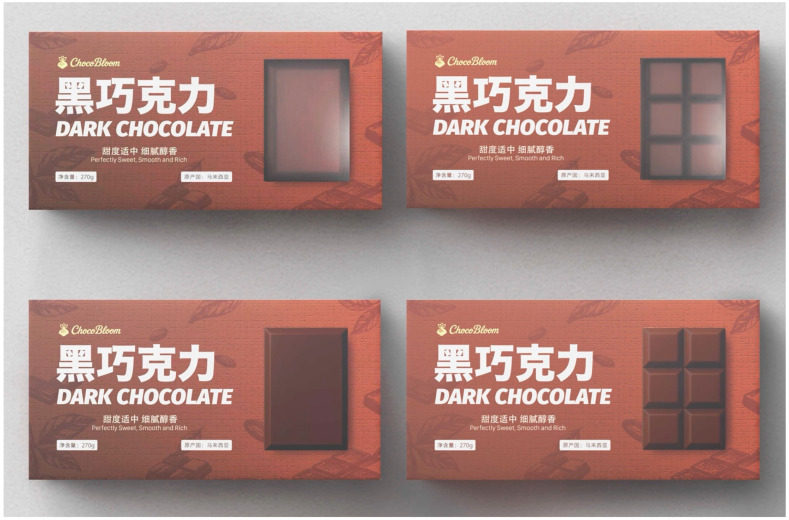
Experimental stimuli: four packaging types based on PVS (transparent window and product image) and FUS (large unit and small unit).

All packaging prototypes were created using a standardized stimulus-development protocol to ensure high internal validity and to remove potential confounds in visual presentation. Four physical packaging prototypes (Figure 1) were produced, representing a 2 (PVS: transparent window and product image) × 2 (FUS: large unit and small unit) design. All other elements, such as brand name, typography, background texture, colors, layout, net weight, and information panels, were kept strictly identical across conditions.

For the PVS manipulation, all chocolate photographs were taken from the same batch of products using a controlled studio setup with fixed shooting conditions. A professional color calibration workflow was applied using an X-Rite ColorChecker system (X-Rite Inc., Grand Rapids, MI, USA) to ensure consistent color accuracy across all images. The calibrated photographs were then used in the product image conditions, which prevented unintended differences in chocolate appearance that could influence visual attention or quality judgments.

**Figure 2:**
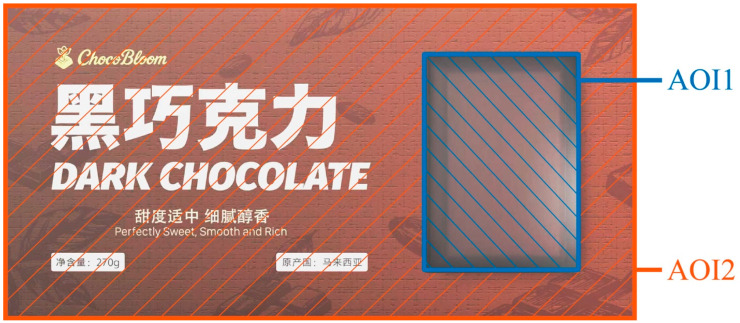
Division of the chocolate packaging into two Areas of Interest (AOI1: product visibility area; AOI2: informational area).

For the FUS manipulation, both the large-unit and small-unit versions were created using chocolate from the same batch. The large-unit version showed one continuous 2 × 3 chocolate block without segmentation. The small-unit version showed six chocolate pieces of the same size arranged in a 2 × 3 layout, with clear segmentation lines added to display the unit separation. To avoid extra visual cues such as interior blank space, product density, or fullness of the package, both unit-size versions occupied exactly the same visual area on the package. As shown in Figure 2, this visual area was defined as AOI 1, which represents the main focus area for consumers’ product perception. The area outside AOI1 was defined as AOI2, the “product information area”, which includes text, brand elements, and other visual information related to brand perception and design features.

All four packaging versions were printed using a high-resolution CMYK digital inkjet printing process on 350 g white cardboard to ensure consistency in color reproduction and surface texture across conditions. The same printing device and material specifications were applied to all prototypes to avoid unintended visual variation between experimental stimuli. To ensure the realism and construct validity of the stimuli, a panel of experts in marketing, consumer behavior, and visual design reviewed the packaging prototypes. They evaluated the clarity and realism of the package presentation. Minor adjustments were made based on their feedback, such as balancing spacing in the small-unit condition and adjusting the window proportion.

### 2.3. Participants

We recruited 192 participants from four universities in Jinan using a voluntary sampling method. Each participant completed a demographic questionnaire, which was used to identify their academic background. Students majoring in marketing, psychology, or design were considered to possess prior knowledge of the experimental stimuli. Accordingly, 32 participants from these related fields were excluded to eliminate potential bias arising from previous experience and domain-specific knowledge, which could otherwise distort visual cognitive performance [34]. This exclusion enabled us to capture more general cognitive responses to visual stimuli and thereby enhance the external validity and generalizability of the findings.

During data collection, participants with low eye-tracking sampling rates (below 90%) were also excluded. Consequently, valid data from 160 participants were retained for analysis, including 68 males and 92 females. All participants had normal or corrected-to-normal vision (visual acuity between 1.0 and 1.5) and were free from color blindness or color weakness, minimizing potential visual biases during the experiment. Prior to the experiment, all participants provided written informed consent.

A priori power analysis was conducted using G*Power 3.1 [35]. Assuming a medium effect size (f = 0.25), α = 0.05, and power = 0.80 for a 2 × 2 between-subjects ANOVA, the required minimum sample size was 128 participants. The final valid sample of 160 participants exceeded this threshold.

### 2.4. Instrument

The experiment was conducted in a closed and soundproof laboratory to ensure environmental control and precision in data collection. During the preparation stage, the finalized experimental stimuli were placed in opaque boxes by researchers so that participants were unaware of any information regarding the stimuli prior to the experiment. The study employed the Tobii Glasses Pro 2 eye-tracking device (Tobii Pro AB, Stockholm, Sweden), which enables accurate capture of participants’ eye-movement data [36]. Two key eye-tracking indicators were collected: FD (the total time participants spent fixating on a specific area or object) and TFF (the time elapsed from the onset of the stimulus to the participant’s first fixation on that area or object).

The questionnaire items used in this study were adapted from previously validated measures of related variables, including Expected Tastiness, Expected Quality, and Purchase Intention. These items have been adopted in prior empirical studies (Table 1) and were used to construct the measurement instrument for this research. All items were rated on a seven-point Likert scale (1 = strongly disagree, 7 = strongly agree). As this study was conducted in China, all questionnaire contents were translated into Chinese and reviewed bilingually to ensure linguistic clarity and conceptual equivalence.

### 2.5. Data Collection Procedure

Before the experiment began, participants received detailed instructions and explanations about the research procedures. The preliminary briefing aimed to ensure that participants fully understood the experimental process, thereby enhancing the reliability and validity of the study. Research assistants helped participants wear the eye-tracking devices, which were carefully calibrated to ensure accurate data collection and to accommodate individual differences in eye physiology, ensuring that subsequent data accurately reflected participants’ visual attention. During the experiment, no specific tasks were assigned, and participants were allowed to freely view the chocolate packaging stimuli.

**Figure 3:**
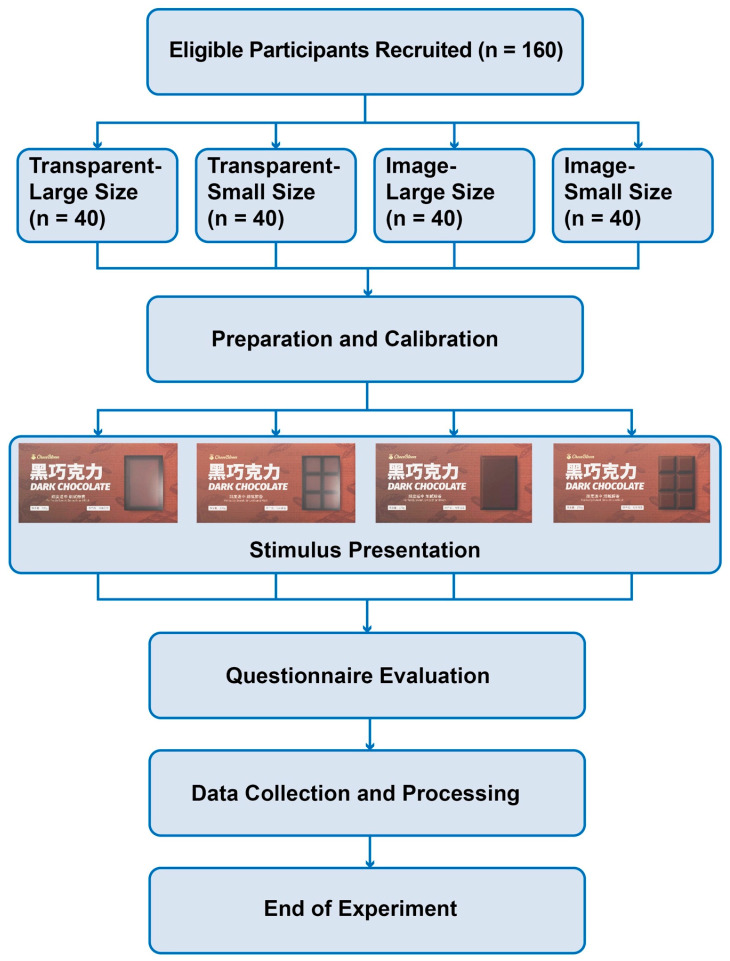
Experimental procedure demonstration.

The experiment adopted a 2 × 2 between-subjects quasi-experimental design, with participants randomly assigned to four experimental groups. Each participant viewed and evaluated only one packaging stimulus to ensure the independence of visual attention and subjective evaluation, while avoiding visual fatigue and learning effects that could bias the results. The experimental procedure is illustrated in Figure 3.

Participants were seated in front of a screen and instructed to read the experimental guidelines displayed on the board. To confirm understanding, they signaled their readiness by raising their hands to the research assistants. The assistants then took a stimulus out of the box and placed it at a fixed position on the table for the participant to view. During observation, participants completed the rating scale corresponding to the stimulus.

This dual-task procedure of observation and evaluation was designed to simulate real-world conditions, where the human brain simultaneously processes visual information and forms judgments [39]. Participants rated the stimuli using the provided scale while carefully observing the images. Upon completion, they raised their hands again to indicate the end of the current experimental session. This tightly controlled procedure ensured the accuracy and consistency of data collection.

### 2.6. Data Analysis

To ensure the accuracy and reliability of the research results, systematic statistical analysis methods were employed. Eye-tracking data were recorded using Tobii Pro Glasses Controller software (version 1.114, Tobii Pro AB, Stockholm, Sweden) and processed and exported through Tobii Pro Lab software (version 25.7, Tobii Pro AB, Stockholm, Sweden). The numerical data for Expected Tastiness, Expected Quality, and Purchase Intention were manually entered into Microsoft Excel to ensure accurate documentation of self-reported ratings and to prepare the data for subsequent statistical analyses. All statistical analyses were conducted using SPSS for Windows, version 26.0. Descriptive statistics were used to summarize the quantitative results under the four different stimulus conditions. These descriptive statistics provided an overview of the dataset, including measures of central tendency (mean, median) and dispersion (standard deviation), which helped to identify overall trends and variations within the data [40].

To examine the effects of the independent variables, analyses of main effects and interaction effects were performed. These analyses were used to determine the primary and combined influences of packaging design variations on the dependent variables, Expected Tastiness, Expected Quality, Purchase Intention, and visual behavior indicators (FD and TFF). Main effect analyses assessed the independent influence of each factor, helping to clarify how PVS and FUS individually affected participants’ visual behavior and subjective evaluations. Interaction effect analyses further examined whether the effect of one independent variable depended on the level of the other, offering deeper insights into the combined effects of different packaging designs [41].

When significant interactions were found, simple effect analyses were conducted to further explore these interactions in detail. This stepwise approach allowed the examination of how one independent variable affected the dependent variables at specific levels of the other variable, thereby clarifying subtle differences in how various factors influenced visual behavior and subjective evaluations. All statistical tests were conducted at a significance level of *p* < 0.05.

## 3. Results

A total of 160 eligible participants (92 women and 68 men) with a mean age of 20.88 (SD = 2.06) were recruited for this study. Participants were randomly assigned to four groups (40 participants each) using a 2 × 2 between-subjects quasi-experimental design (PVS × FUS). Participants in each group viewed only one package for 30 s.

### 3.1. Fixation Duration

**Figure 4:**
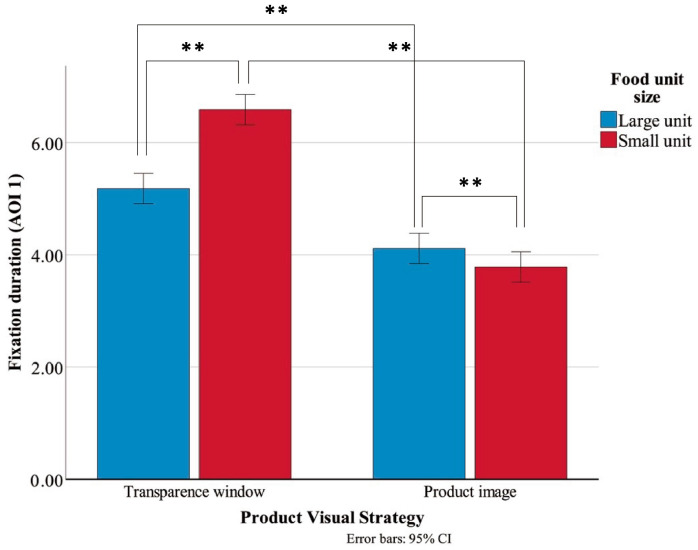
Interaction effect of FD on AOI1. Error bars represent 95% confidence intervals. ** *p* < 0.01.

The descriptive statistics for FD on AOI1 are presented in Table 2. The main effect of PVS was significant, F(1, 156) = 201.593, *p* < 0.001, η^2^ = 0.564, indicating that the transparent window condition (M = 5.89, SD = 1.17) resulted in significantly longer FD than the product image condition (M = 3.95, SD = 0.79). The main effect of FUS was also significant, F(1, 156) = 15.537, *p* < 0.001, η^2^ = 0.091, showing that participants fixated significantly longer on small-unit products (M = 5.19, SD = 1.63) than on large-unit products (M = 4.65, SD = 1.05). Moreover, the interaction effect between PVS and FUS was significant, F(1, 156) = 40.576, *p* < 0.001, η^2^ = 0.206, as illustrated in Figure 4.

Further simple effect analyses were conducted for FD on AOI1 (Table 3). The results showed that, within both large-unit and small-unit product conditions, the transparent window always has a significantly longer FD than the product image. Within the transparent window condition, FD for large-unit products was significantly shorter than for small-unit products, whereas within the product image condition, FD for large-unit products was significantly longer than for small-unit products.

**Figure 5:**
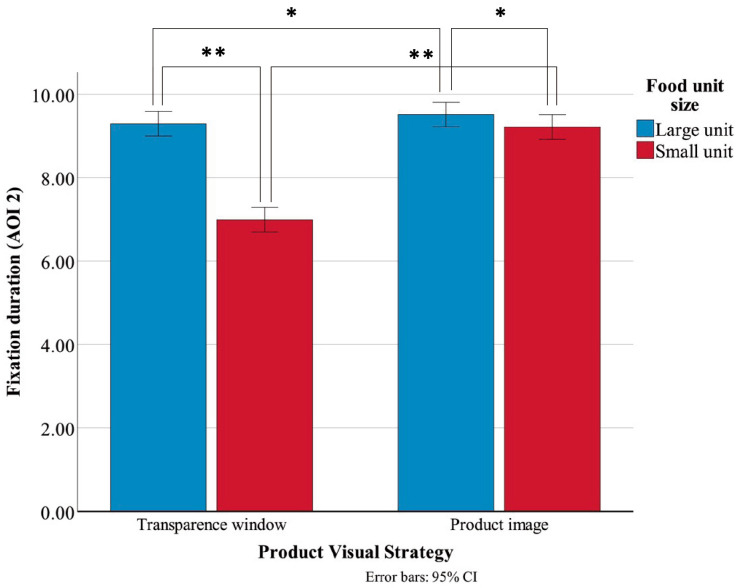
Interaction effect of FD on AOI2. Error bars represent 95% confidence intervals. * *p* < 0.05; ** *p* < 0.01.

The descriptive statistics for FD on AOI2 are summarized in Table 4. A significant main effect of PVS was observed, F(1, 156) = 66.719, *p* < 0.001, η^2^ = 0.300, indicating that transparent window packaging (M = 8.14, SD = 1.51) generated significantly shorter FD than product image packaging (M = 9.36, SD = 0.93). The main effect of FUS was also significant, F(1, 156) = 75.111, *p* < 0.001, η^2^ = 0.325, showing that participants fixated longer on large-unit products (M = 9.40, SD = 0.93) than on small-unit products (M = 8.10, SD = 1.48). Moreover, a significant PVS × FUS interaction was found, F(1, 156) = 44.590, *p* < 0.001, η^2^ = 0.222 (Figure 5), suggesting that the influence of PVS on FD varied according to product size.

To further interpret this interaction, simple effect analyses were performed (Table 5). The results indicated that, for large-unit products, there was no significant difference in FD between the transparent window and product image conditions. However, for small-unit products, FD in the transparent window condition was significantly shorter than in the product image condition. Within the transparent window condition, FD for large-unit products was significantly longer than for small-unit products, whereas within the product image condition, there was no significant difference in FD between large-unit and small-unit products.

### 3.2. Time to First Fixation

**Figure 6:**
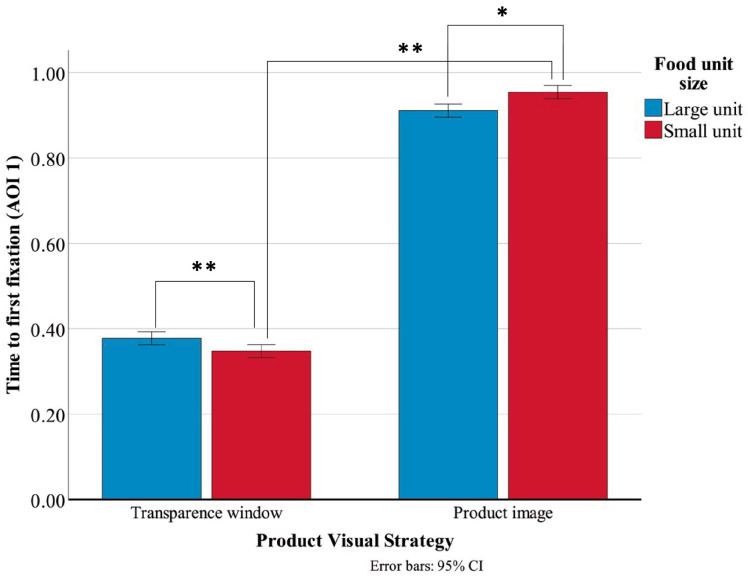
Interaction effect of TFF on AOI1. Error bars represent 95% confidence intervals. * *p* < 0.05; ** *p* < 0.01.

The descriptive statistics for TFF on AOI1 are reported in Table 6. The main effect of PVS was significant, F(1, 156) = 542.561, *p* < 0.001, η^2^ = 0.972, indicating that the transparent window condition (M = 0.36, SD = 0.04) resulted in significantly shorter TFF than the product image condition (M = 0.93, SD = 0.06). The main effect of FUS was not significant (*p* = 0.403). However, the interaction effect between PVS and FUS was significant (Figure 6), F(1, 156) = 22.517, *p* < 0.001, η^2^ = 0.126.

Further simple effect analyses were conducted for TFF on AOI1 (Table 7). The results revealed that, across both large-unit and small-unit product conditions, transparent window packages consistently produced significantly shorter TFF than product image packages. Within the transparent window condition, large-unit products elicited significantly longer TFF than small-unit products. Conversely, within the product image condition, large-unit products showed significantly shorter TFF than small-unit products.

The descriptive statistics for TFF on AOI2 are presented in Table 8. The main effect of PVS was significant, F(1, 156) = 4.684, *p* = 0.032, η^2^ = 0.029, indicating that transparent window packages (M = 1.22, SD = 0.41) produced significantly longer TFF than product image packages (M = 1.08, SD = 0.42). The main effect of FUS was not significant (*p* = 0.321). The interaction effect between PVS and FUS was also not significant (*p* = 0.018).

### 3.3. Expected Tastiness

The descriptive statistics for Expected Tastiness are shown in Table 9. The main effect of PVS was significant, F(1, 156) = 31.859, *p* < 0.001, η^2^ = 0.170, indicating that the transparent window condition (M = 5.41, SD = 1.00) resulted in significantly higher expected tastiness ratings than the product image condition (M = 4.53, SD = 0.99). The main effect of FUS was not significant (*p* = 0.958). The interaction effect between PVS and FUS was also not significant (*p* = 0.062).

### 3.4. Expected Quality

As shown in Table 10, Expected Quality was significantly influenced by PVS, F(1, 156) = 39.302, *p* < 0.001, η^2^ = 0.201. Participants perceived the transparent window packages (M = 4.96, SD = 0.84) as having higher expected quality than the product image packages (M = 4.00, SD = 1.08). In contrast, no significant effects were found for Food Unit Size (FUS) (*p* = 0.330) or for the interaction between PVS and FUS (*p* = 0.516).

### 3.5. Purchase Intention

**Figure 7:**
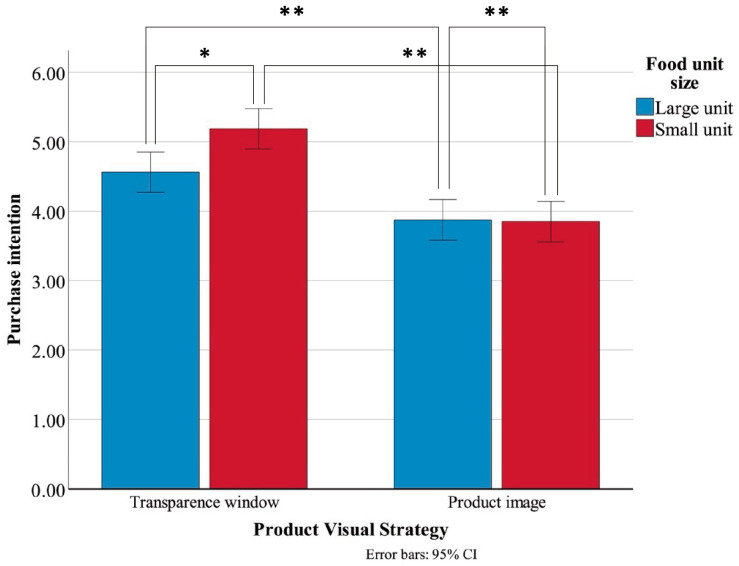
Interaction effect of Purchase Intention. Error bars represent 95% confidence intervals. * *p* < 0.05; ** *p* < 0.01.

As displayed in Table 11, participants reported higher Purchase Intention scores for packages with a transparent window (M = 4.88, SD = 1.05) compared to those with a product image (M = 3.86, SD = 0.85), F(1, 156) = 47.067, *p* < 0.001, η^2^ = 0. 232, indicating a strong main effect of PVS. Moreover, a significant main effect of FUS was also observed, F(1, 156) = 4.132, *p* = 0.044, η^2^ = 0.026, with large-unit products (M = 4.22, SD = 1.05) eliciting lower purchase intentions than small-unit products (M = 4.52, SD = 1.09). Furthermore, the interaction between PVS and FUS reached significance, F(1, 156) = 4.849, *p* = 0.029, η^2^ = 0.030, as illustrated in Figure 7, suggesting that the effect of packaging transparency on purchase intention varied depending on product unit size.

Further simple effect analyses for Purchase Intention were conducted (Table 12). The results demonstrated that, across both large-unit and small-unit product conditions, participants reported significantly higher purchase intentions for the transparent window packages than for the product image packages. Within the transparent window condition, the purchase intention of large-unit products is significantly lower than that of small-unit products, while in the product image condition, there is no significant difference in purchase intention between large-unit and small-unit products.

## 4. Discussion

This study examined how food packaging PVS and FUS jointly influence consumers’ visual attention, perceptual evaluations, and purchase intentions. Overall, the findings revealed a significant advantage of transparent packaging, which effectively attracted more visual attention to the product area, enhanced consumers’ perceived tastiness and quality, and ultimately increased their purchase intentions. Eye-tracking results showed that transparent packaging significantly increased FD on AOI1 and shortened the TFF in that area. This indicates that consumers’ attention and interest toward the visible product were faster and stronger. Moreover, a significant interaction effect between PVS and FUS was observed; that is, under the transparent condition, small-unit products attracted more visual attention and higher purchase intentions, while this effect disappeared under the product-image condition. Consistent with these eye-tracking results, the subjective evaluations further confirmed that the transparent visual strategy enhanced expected tastiness, perceived quality, and purchase intention. Notably, there was a significant interaction between PVS and FUS: transparent packaging with a small-unit product generated the highest purchase intention among participants, but this effect was absent under the product-image condition. These results suggest that product size only has an impact when the product is visible.

The eye-tracking results revealed a clear difference in the distribution of visual attention between AOI1 and AOI2 on the product packaging. Transparent packaging attracted significantly longer FD and shorter TFF on AOI1, while the attention in the AOI2 area was relatively reduced. This indicates that transparent packaging effectively directs participants’ attention to the product itself, namely, the internal cues. This finding can be explained by the Cue Utilization Theory. When intrinsic cues (the product itself visible through transparent packaging) are easily captured, consumers rely less on extrinsic cues (product information presented in AOI2) when making evaluations [42,43,44]. In other words, the transparent window design provides an intrinsic cue that attracts consumers’ attention and facilitates their subjective product perception. Consequently, visual attention becomes concentrated on the product due to intrinsic cue exposure, leading to a shorter initial exposure time (TFF) and longer FD. The change in visual attention during this process may further stimulate consumers’ impulsive buying behavior, as impulse buying is often driven by external visual stimulation and timely sensory responses [45,46].

These results are consistent with many previous studies showing that transparent window designs increase product visibility and direct consumers’ visual attention toward the food itself, thereby enhancing their perceptual evaluations [14,18,47]. Different from previous studies that examined the transparency effect alone, this study conducted a 2 × 2 between-subjects quasi-experimental design combined with eye-tracking technology to explore the joint effects of PVS and FUS on consumers’ visual attention and subjective perception. By integrating objective visual attention metrics with subjective evaluations, this study provides a more comprehensive explanation of the interaction mechanism between visual cues and consumer responses. In summary, this attention shift highlights the crucial role of visible visual strategies in packaging design. By enhancing product visibility, designers can strategically guide consumers’ gaze toward the product, elevate perceptual evaluations, and ultimately influence purchase decisions.

A significant interaction was observed between PVS and FUS. Specifically, under the transparent condition, small-unit products attracted longer FD and higher purchase intentions than large-unit products. However, under the product-image condition, this advantage of small-unit products disappeared—both large and small units exhibited similar levels of visual attention and evaluation. These findings are consistent with the Limited Capacity Model of Attention, which posits that consumers’ attentional resources are limited and are preferentially allocated to stimuli perceived as more informative or novel [48,49]. The transparent window design functions both as an intrinsic cue, providing direct exposure to the actual product, and as an extrinsic cue, offering a novel visual presentation that stimulates consumer interest. Consequently, the visual salience of transparent packaging can be amplified for both large and small unit sizes, leading to longer gazes and better taste and quality perceptions.

Furthermore, these results can be explained by the perspective of Deng and Srinivasan [21], who pointed out that small-unit products often elicit more positive consumer responses because they weaken self-monitoring effects and activate reward-related processes associated with hedonic motivation [50,51]. The present findings suggest that this psychological mechanism operates primarily when the product is visually accessible. When transparent packaging reveals the intrinsic cue, the small-unit product is perceived as less indulgent or more acceptable, which subconsciously encourages higher attentional engagement and more favorable subjective evaluations. In contrast, when the product itself is concealed (product-image condition), consumers rely more heavily on extrinsic cues. In this case, self-monitoring tendencies are reactivated, and the advantage of small-unit products diminishes due to the lack of direct product visibility. In summary, the coordination between PVS and FUS plays a crucial role in enhancing visual appeal and promoting subjective evaluations and purchase intentions. Designers should strategically balance product visibility and product size to create packaging that not only captures attention but also drives behavioral responses in purchase decision-making.

The subjective evaluation results of product transparency were consistent with previous studies, indicating that the packaging’s PVS influences not only consumers’ product perceptions but also their purchase intentions [5,14,19]. In the present study, all transparent packaging conditions significantly enhanced perceived tastiness, perceived quality, and purchase intention compared with product-image packaging, whereas the subjective effect of FUS was non-significant except for purchase intention. These findings can be interpreted through the lens of Processing Fluency Theory [52]. Transparent packaging exposes the product itself, making visual information easier to process. During this process, consumers experience psychological fluency and transform this sense of ease and familiarity into positive affect and trust in product quality, which in turn leads to more favorable evaluations and stronger purchase intentions [53,54,55]. In other words, processing fluency serves as an emotional bridge between PVS and behavioral responses. In contrast, the effect of product size was significant only for purchase intention but not for perceptual evaluations. This may be because the visual salience of transparent packaging occupies the majority of attentional resources, diminishing the influence of size-related cues. Moreover, product size may primarily affect behavioral intentions through motivational and reward-related mechanisms rather than direct perceptual evaluations. Overall, visual visibility exerts a more direct and stronger influence on consumers’ perceptual responses than product size.

Beyond explaining the specific empirical findings, the present study offers several theoretical contributions. First, by integrating eye-tracking measures with packaging design variables, this research extends the application of the Eye–Mind Hypothesis to consumer decision-making contexts. The systematic differences in fixation duration and attentional orientation across packaging conditions provide evidence that variations in packaging visibility are reflected in observable differences in visual processing patterns. Second, the observed interaction between product visual strategy and food unit size enriches Cue Utilization Theory by showing that intrinsic and extrinsic cues may differentially influence attention allocation depending on their visual accessibility. When intrinsic cues are made directly visible through transparent packaging, they attract greater visual attention and are associated with more favorable evaluations. Third, by distinguishing between early attentional orientation (TFF) and sustained visual engagement (FD), this study refines the understanding of how different components of visual attention correspond to consumer responses in packaging contexts. Together, these findings position the study within both packaging research and broader visual attention literature.

From a practical standpoint, the findings suggest that packaging strategies should consider the interaction between product visibility and product structure. Transparent window designs appear particularly effective for products composed of smaller consumption units, where visual exposure is associated with higher attentional engagement and more favorable consumer evaluations. For larger-unit products, increasing transparency may not generate comparable differences in visual attention patterns. Therefore, designers may benefit from aligning visual strategy with product characteristics rather than uniformly increasing transparency. These findings provide guidance for packaging decisions grounded in observed visual attention patterns.

## 5. Conclusions

This study purposed to investigate the effects of PVS and FUS on consumers’ visual attention, perceptual evaluations, and purchase intentions. By combining eye-tracking techniques with a subjective questionnaire, the study measured participants’ FD and TFF on clearly defined packaging areas, while simultaneously collecting self-reported ratings of expected tastiness, perceived quality, and purchase intention. The findings highlight that transparent window designs, as a form of PVS, exert significant positive effects on both visual attention and perceptual outcomes. In contrast, the influence of FUS was limited, contributing only marginally to purchase intention. Notably, the advantage of small-unit products emerged only under the transparent condition, whereas under the low-visibility product-image condition, the size effect disappeared.

This study still has several limitations despite its rigorous design. First, the sample consisted primarily of Chinese university students, which may limit the generalizability of the findings to other age groups or cultural contexts. Subsequent research ought to incorporate participants from a broader range of demographic backgrounds to improve external validity. Second, the controlled laboratory setting may not fully capture the complexity of real-world retail environments. In actual supermarkets, consumers are exposed to multiple competing brands, varied shelf placements (such as eye-level and lower shelves), price labels, promotional signage, and surrounding visual clutter. These contextual factors may influence attention allocation and purchase decisions in ways that differ from controlled experimental conditions. Therefore, the magnitude of the observed effects may vary in natural shopping contexts. In addition, although the stimuli in this study underwent expert review and pilot optimization to ensure visual consistency and effective manipulation, the presentation of the product in the transparent window condition may still differ slightly from commercial packaging used in the marketplace. While such simplifications are common in experimental research to maintain internal validity, future studies should attempt to replicate these findings using market-ready packaging designs. Future research could address these limitations by employing more diverse samples, testing a broader range of product categories, and utilizing immersive technologies such as virtual reality or augmented reality to simulate realistic shopping environments. Additionally, combining eye-tracking with qualitative methods may provide deeper insights into how PVS and FUS influence consumer perception and decision-making.

## Figures and Tables

**Table 1:** Measurement items for Expected Tastiness, Expected Quality, and Purchase Intention.

Item No.	Variable	Question
1	Expected Tastiness [36,37]	How would you rate the likely taste of the product in this packaging?
2		If you had to guess, how delicious might the product in this packaging be?
3	Expected Quality [14,38]	What quality would you expect this product to be?
4		Does the product appear to be of good quality?
5	Purchase Intention [24]	I consider buying this product.
6		There is a strong likelihood that I will buy this product.

Note: Items were rated on a 7-point Likert scale (1 = strongly disagree; 7 = strongly agree). Expected Tastiness and Expected Quality were measured using two items each. Purchase Intention was measured using two items.

**Table 2:** Descriptive statistics of FD on AOI1.

PVS	FUS	M ± SD (s)	N
Transparent window	Large unit	5.18 ± 0.97	40
	Small unit	6.59 ± 0.90	40
	Total	5.89 ± 1.17	80
Product image	Large unit	4.11 ± 0.83	40
	Small unit	3.78 ± 0.73	40
	Total	3.95 ± 0.79	80
Total	Large unit	4.65 ± 1.05	80
	Small unit	5.19 ± 1.63	80
	Total	4.92 ± 1.39	160

Note: PVS = Product Visual Strategy; FUS = Food Unit Size; AOI = Area of Interest; Values are presented as M ± SD; N = sample size.

**Table 3:** Results of the simple effect of FD on AOI1.

Variable		I	J	Mean Difference (I − J)	F	*p*
FUS	Large unit	Transparent window	Product image	1.07	30.642	<0.001
	Small unit	Transparent window	Product image	2.81	211.528	<0.001
PVS	Transparent window	Large unit	Small unit	−1.41	53.165	<0.001
	Product image	Large unit	Small unit	0.33	2.948	<0.001

Note: I and J indicate the compared conditions. Mean Difference (I − J) represents the difference between group means. F and *p* values are derived from two-way ANOVA analyses.

**Table 4:** Descriptive statistics of FD on AOI2.

PVS	FUS	M ± SD (s)	N
Transparent window	Large unit	9.29 ± 0.94	40
	Small unit	6.99 ± 1.01	40
	Total	8.14 ± 1.51	80
Product image	Large unit	9.51 ± 0.92	40
	Small unit	9.22 ± 0.93	40
	Total	9.36 ± 0.93	80
Total	Large unit	9.40 ± 0.93	80
	Small unit	8.10 ± 1.48	80
	Total	8.75 ± 1.39	160

Note: PVS = Product Visual Strategy; FUS = Food Unit Size; AOI = Area of Interest; Values are presented as M ± SD; N = sample size.

**Table 5:** Results of the simple effect of FD on AOI2.

Variable		I	J	Mean Difference (I − J)	F	*p*
FUS	Large unit	Transparent window	Product image	−0.22	1.111	0.294
	Small unit	Transparent window	Product image	−2.23	110.198	<0.001
PVS	Transparent window	Large unit	Small unit	2.30	117.723	<0.001
	Product image	Large unit	Small unit	0.30	1.978	0.013

Note: I and J indicate the compared conditions. Mean Difference (I − J) represents the difference between group means. F and *p* values are derived from two-way ANOVA analyses.

**Table 6:** Descriptive statistics of TFF on AOI1.

PVS	FUS	M ± SD (s)	N
Transparent window	Large unit	0.38 ± 0.04	40
	Small unit	0.35 ± 0.05	40
	Total	0.36 ± 0.04	80
Product image	Large unit	0.91 ± 0.05	40
	Small unit	0.95 ± 0.06	40
	Total	0.93 ± 0.06	80
Total	Large unit	0.64 ± 0.27	80
	Small unit	0.65 ± 0.31	80
	Total	0.65 ± 0.29	160

Note: PVS = Product Visual Strategy; FUS = Food Unit Size; AOI = Area of Interest; Values are presented as M ± SD; N = sample size.

**Table 7:** Results of the simple effect of TFF on AOI1.

Variable		I	J	Mean Difference (I − J)	F	*p*
FUS	Large unit	Transparent window	Product image	−0.53	2372.644	<0.001
	Small unit	Transparent window	Product image	−0.61	3071.434	<0.001
PVS	Transparent window	Large unit	Small unit	0.03	7.628	0.006
	Product image	Large unit	Small unit	−0.043	15.593	<0.001

Note: I and J indicate the compared conditions. Mean Difference (I − J) represents the difference between group means. F and *p* values are derived from two-way ANOVA analyses.

**Table 8:** Descriptive statistics of TFF on AOI2.

PVS	FUS	M ± SD (s)	N
Transparent window	Large unit	1.13 ± 0.35	40
	Small unit	1.31 ± 0.45	40
	Total	1.22 ± 0.41	80
Product image	Large unit	1.10 ± 0.42	40
	Small unit	1.06 ± 0.42	40
	Total	1.08 ± 0.42	80
Total	Large unit	1.12 ± 0.38	80
	Small unit	1.18 ± 0.45	80
	Total	1.15 ± 0.42	160

Note: PVS = Product Visual Strategy; FUS = Food Unit Size; AOI = Area of Interest; Values are presented as M ± SD; N = sample size.

**Table 9:** Descriptive statistics of Expected Tastiness.

PVS	FUS	M ± SD (s)	N
Transparent window	Large unit	5.26 ± 0.95	40
	Small unit	5.56 ± 1.03	40
	Total	5.41 ± 1.00	80
Product image	Large unit	4.68 ± 1.01	40
	Small unit	4.39 ± 0.96	40
	Total	4.53 ± 0.99	80
Total	Large unit	4.97 ± 1.02	80
	Small unit	4.98 ± 1.15	80
	Total	4.97 ± 1.08	160

Note: PVS = Product Visual Strategy; FUS = Food Unit Size; AOI = Area of Interest; Values are presented as M ± SD; N = sample size.

**Table 10:** Descriptive statistics of Expected Quality.

PVS	FUS	M ± SD (s)	N
Transparent window	Large unit	4.84 ± 0.83	40
	Small unit	5.09 ± 0.85	40
	Total	4.96 ± 0.84	80
Product image	Large unit	3.98 ± 1.20	40
	Small unit	4.03 ± 0.97	40
	Total	4.00 ± 1.08	80
Total	Large unit	4.41 ± 1.11	80
	Small unit	4.56 ± 1.05	80
	Total	4.48 ± 1.08	160

Note: PVS = Product Visual Strategy; FUS = Food Unit Size; AOI = Area of Interest; Values are presented as M ± SD; N = sample size.

**Table 11:** Descriptive statistics of Purchase Intention.

PVS	FUS	M ± SD (s)	N
Transparent window	Large unit	4.56 ± 1.07	40
	Small unit	5.19 ± 0.94	40
	Total	4.88 ± 1.05	80
Product image	Large unit	3.88 ± 0.93	40
	Small unit	3.85 ± 0.77	40
	Total	3.86 ± 0.85	80
Total	Large unit	4.22 ± 1.05	80
	Small unit	4.52 ± 1.09	80
	Total	4.37 ± 1.08	160

Note: PVS = Product Visual Strategy; FUS = Food Unit Size; AOI = Area of Interest; Values are presented as M ± SD; N = sample size.

**Table 12:** Results of the simple effect of Purchase Intention.

Variable		I	J	Mean Difference (I − J)	F	*p*
FUS	Large unit	Transparent window	Product image	0.69	10.850	0.001
	Small unit	Transparent window	Product image	1.33	41.066	<0.001
PVS	Transparent window	Large unit	Small unit	−0.63	8.967	0.003
	Product image	Large unit	Small unit	0.03	0.014	0.905

Note: I and J indicate the compared conditions. Mean Difference (I − J) represents the difference between group means. F and *p* values are derived from two-way ANOVA analyses.

## Data Availability

The data supporting the findings of this study are available from the corresponding author upon reasonable request. Due to privacy and ethical restrictions, the dataset is not publicly available.

## Abbreviations

The following abbreviations are used in this manuscript:

PVS	Product Visual Strategy
FUS	Food Unit Size
FD	Fixation Duration
TFF	Time to First Fixation
AOI	Area of Interest
